# Ash-free dry mass values for northcentral USA caddisflies (Insecta, Trichoptera)

**DOI:** 10.3897/zookeys.951.49790

**Published:** 2020-07-22

**Authors:** David C. Houghton, Ryan Lardner

**Affiliations:** 1 Department of Biology, Hillsdale College, 33 East College Street, Hillsdale, MI 49242, USA Hillsdale College Hillsdale United States of America

**Keywords:** ash-free dry mass, biomass, caddisfly, Great Lakes, organic, Trichoptera

## Abstract

Ash-free dry mass (AFDM) values are presented for the adult stage of 63 caddisfly species commonly found throughout the northcentral US. Weights ranged from 0.01 mg for the smallest species to 7.22 mg for the largest. These values represent the first published data on the AFDM of the adult stage of Trichoptera, and can be used in other studies for more precise assessments of stream conditions without destruction of specimens. This increased precision is demonstrated herein by re-analyzing a previously published data set.

## Introduction

The organic biomass of organisms is one of the most important quantifiable variables in ecological studies. Measurements of biomass are informative about ecosystem production, metabolism, food web ecology, and the overall health and biotic integrity of the community (Enquist and Niklas 2001; Gruner et al. 2008; Eklöf et al. 2017). For aquatic ecosystems, biomass measurements are also indicative of the relative contribution of different functional feeding groups (FFGs), which can be used to assess ecosystem continuity, types and availability of organic carbon, and anthropogenic disturbance (Vannote et al. 1980; Barbour et al. 1999).

There are several measurements used to express the biomass of organisms, including wet mass, dry mass, and ash-free dry mass (AFDM). To determine AFDM, specimens are incinerated at temperatures high enough to volatilize organic tissue but not inorganic tissue. The difference between pre-incineration and post-incineration weights reflects the mass of the organic tissue volatized. AFDM is considered the most accurate measurement of biomass since it encompasses the biologically active tissue (Eklöf et al. 2017).

Various parameters of immature aquatic insect assemblages, including their AFDM, have been used for many years to assess the functioning and biotic integrity of aquatic ecosystems. Some challenges to using the immature stage, such as the difficulty of sampling all aquatic microhabitats representatively and identifying specimens to the species level, can be alleviated by using the winged adult stage, particularly that of taxonomically and ecologically diverse groups such as the caddisflies (Trichoptera) (Gerth and Herily 2006; Chessman et al. 2007; Cao and Hawkins 2011; Houghton et al. 2011). Assemblages of caddisfly adults, particularly the relative abundance of specimens within different FFGs, have been shown in several studies to be indicative of stream conditions (Dohet 2002; Houghton 2007; Blinn and Ruiter 2013; Houghton et al. 2018). Such studies, however, treated all specimens equally and did not reflect the differences in biomass between different species. Since the largest caddisfly species are >100× heavier than the smallest species, not accounting for this difference results in a loss of precision. Because measurements of FFG biomass directly relate to the biomass of available carbon sources and, thus, habitat differences, increasing precision in these FFG biomass measurements is of substantial importance.

Due to the necessity of maintaining museum collections of the taxonomically important caddisfly adults, most researchers are understandably reluctant to destroy them in order to obtain AFDM values. Indeed, while many studies have published data on caddisfly larvae (Johnston and Cunjak 1999), we have been unable to find a single one measuring the AFDM of the adult stage, although several have reported dry mass (Svensson 1975; Peterson 1989; Wagner 2002; Wagner 2005; Jannot et al. 2007) or wet mass (Wallace and Howard 1992). The purpose of this study, therefore, was to determine and publish AFDM values of common and abundant caddisfly species in our collection for future ecological studies using adult caddisflies.

## Materials and methods

We have been collecting caddisfly adults in the northcentral US since 2000, mostly utilizing an 8-watt ultraviolet light placed over a white pan filled with 80% EtOH. Such devices can capture 1000s of specimens during a single evening of heavy flight activity. Collected specimens are preserved in 80% EtOH for long-term storage, which limits decomposition and loss of organic biomass over time (Wetzel et al. 2005).

**Figure 1.**
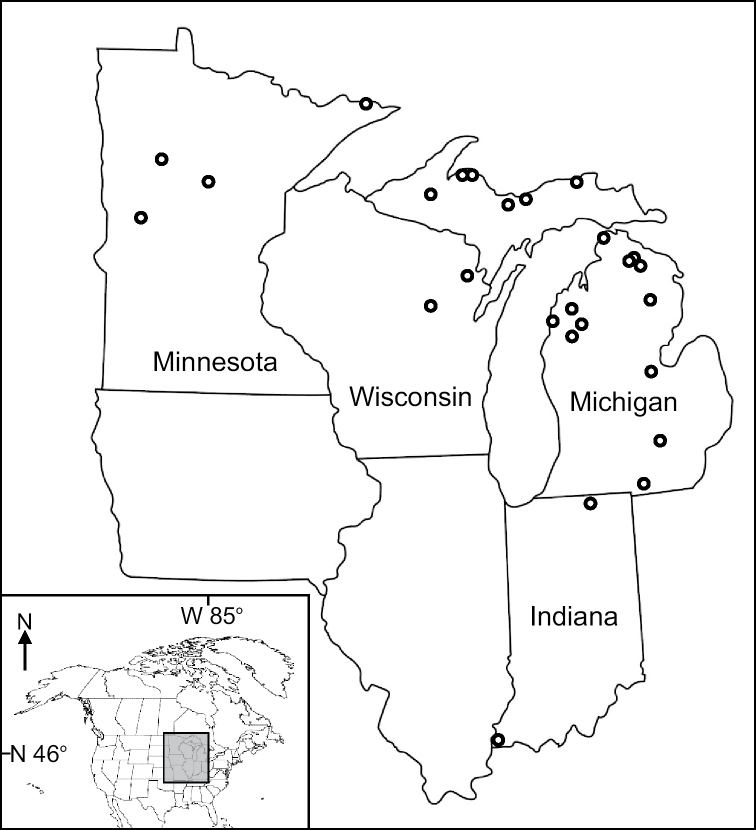
The northcentral US, showing localities from where our AFDM-determined specimens were collected.

Species were chosen for biomass determination largely due to practical considerations. The weight of single specimens of most species is lower than the detection limit of most standard balances. Thus, specimens needed to be weighed in groups of 5 to 500 depending on the size of the species. This limitation meant that we could only determine biomass for abundant species for which we had ample extra specimens. Likewise, the specimen collecting localities that we chose were simply the ones with the most available specimens. Most of these specimens were from Michigan, with some from Indiana, Minnesota, and Wisconsin (Figure 1). Each determined species was from a single collection of a single locality. We generally determined only male specimens, except for some species (e.g., *Psychomyia flavida* Hagen) where females were highly abundant and males were rare. All females were carefully dissected before weighing to confirm they had already oviposited. In no case were both sexes weighed.

To determine organic biomass, specimens of each tested species were taken from their vials of EtOH and placed into pre-dried porcelain crucibles. Crucibles containing the specimens were dried at low heat over a hot plate for several h until all of the EtOH had evaporated and the specimens appeared completely dry. The crucibles and specimens were then further dried for 2 h at 60 °C in a drying oven and then slowly cooled to room temperature before weighing. Crucibles and specimens were then transferred to a muffle furnace and incinerated at 500 °C for 3 h. After cooling to room temperature in the muffle furnace, the resulting material was transferred back to the drying oven, dried for 1 h at 60 °C, cooled to back room temperature, and weighed. AFDM was calculated as the final mass of material remaining after incineration subtracted from the mass of specimens before entering the muffle furnace. Total AFDM per sample divided by the number of specimens in that sample calculated the mean AFDM per specimen. This procedure was repeated 2–5× for each species, depending on how many specimens were available for incineration. Global mean AFDM ± SE for each species was then determined from these data.

## Results

**Figure 2.**
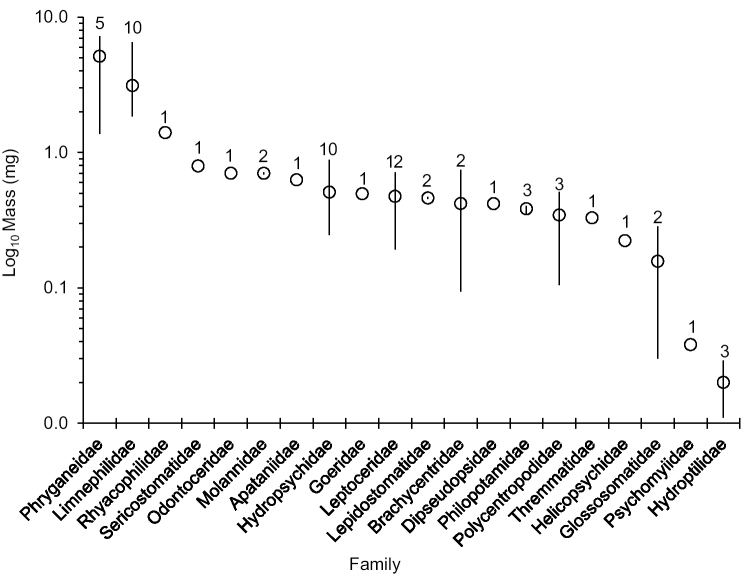
The Log_10_ high, low, and mean biomass values for each of the 20 different caddisfly families measured. Number of species measured within each family above each bar.

Resultant AFDM values are in Table 1. We determined the organic biomass of 63 common caddisfly adults. This total represented 17% (63 of 366) of the known caddisfly species of Indiana, Michigan, Minnesota, and Wisconsin, 58% (47 of 81) of known genera, and all 20 known families. Determined species represented 78% (448589 of 574928) of all caddisfly specimens from the four states in our collection. AFDM values ranged from 7.217 mg for *Ptilostomis semifasciata* (Say) (Phryganeidae) to 0.011 mg for *Orthotrichia aegerfasciella* (Chambers) (Hydroptilidae). Mean familial weight was highest in the Phryganeidae, followed by the Limnephilidae and the Rhyacophilidae. Glossosomatidae, Psychomyiidae, and Hydroptilidae were the lightest families (Figure 2).

**Table 1.** The 63 species of caddisfly adults for which ash-free dry mass (AFDM) (± SE) was determined. Key: Year, year collected. #, number of specimens tested per incineration. *N*, the number of incinerations per species. M/F, whether male or female specimens were measured.

Taxon	Site	Year	#	*N*	M/F	AFDM (mg)	± SE
APATANIIDAE							
*Apatania zonella* (Zetterstedt, 1840)	MI: Lk. Superior, 46.9083, -87.9225	2019	50	3	M	0.628	0.149
BRACHYCENTRIDAE							
*Brachycentrus americanus* (Banks, 1899)	MI: Fairbanks Cr., 44.0481, -85.6586	2014	50	3	M	0.745	0.104
*Micrasema wataga* Ross, 1938	MN: Straight R., 46.8745, -95.0586	2000	300	3	F	0.094	0.026
DIPSEUDOPSIDAE							
*Phylocentropus placidus* (Banks, 1905)	MI: Nunn’s Cr., 46.0572, -84.5639	2010	35	3	M	0.418	0.064
GLOSSOSOMATIDAE							
*Glossosoma nigrior* Banks, 1911	MI: Fairbanks Cr., 44.0481, -85.6586	2011	100	3	F	0.284	0.140
*Protoptila maculata* (Hagen, 1861)	MI: Manistee R., 44.2836, -85.8614	2010	500	3	F	0.030	0.008
GOERIDAE							
*Goera stylata* Ross, 1938	MI: Fairbanks Cr., 44.0481, -85.6586	2011	100	6	M	0.495	0.074
HELICOPSYCHIDAE							
*Helicopsyche borealis* (Hagen, 1861)	MI: Black R., 45.1664, -84.3264	2015	250	6	M	0.223	0.042
HYDROPSYCHIDAE							
*Cheumatopsyche campyla* Ross, 1938	MI: Tittabawasee R., 43.4811, -84.0931	2011	150	6	M	0.346	0.062
*C. speciosa* (Banks, 1904)	MN: Pine R., 46.5717, -94.0281	2000	150	3	F	0.245	0.054
*Diplectrona modesta* Banks, 1908	MI: Fairbanks Cr., 44.0481, -85.6586	2014	50	3	M	0.502	0.071
*Hydropsyche betteni* Ross, 1938	MI: Fairbanks Cr., 44.0481, -85.6586	2014	75	6	M	0.685	0.123
*H. morosa* (Hagen, 1861)	MI: Au Sable R., 44.6599, -84.1292	2011	100	3	M	0.392	0.110
*H. simulans* Ross, 1938	MN: Chippewa R., 45.9408, -95.7383	2000	50	3	M	0.712	0.104
*H. sparna* Ross, 1938	MI: Mountain St., 46.8692, -87.8933	2019	100	3	M	0.452	0.099
*Macrostemum zebratum* (Hagen, 1861)	WI: Peshtigo R., 45.2325, -88.0136	2015	50	3	M	0.884	0.159
*Parapsyche apicalis* (Banks, 1908)	MI: Fairbanks Cr., 44.0481, -85.6586	2011	50	3	M	0.472	0.066
*Potamyia flava* (Hagen, 1861)	IN: Ohio R., 37.7783, -87.9468	2018	200	6	M	0.399	0.072
HYDROPTILIDAE							
*Agraylea multipunctata* Curtis, 1834	IN: Ohio R., 37.7783, -87.9468	2018	500	3	F	0.029	0.004
*Hydroptila xera* Ross, 1938	MI: Two-hearted R., 46.6419, -85.4792	2011	500	3	M	0.017	0.003
*Orthotrichia aegerfasciella* (Chambers, 1873)	MI: Manistee R., 44.2836, -85.8614	2010	500	4	M	0.011	0.003
LEPIDOSTOMATIDAE							
*Lepidostoma bryanti* (Banks, 1908)	MI: Fairbanks Cr., 44.0481, -85.6586	2011	50	6	M	0.452	0.115
*L. togatum* (Hagen, 1861)	MI: Black R., 45.1664, -84.3264	2015	50	6	M	0.469	0.108
LEPTOCERIDAE							
*Ceraclea arielles* (Denning, 1942)	MI: Pine R., 44.1339, -85.6956	2010	150	3	M	0.318	0.054
*C. resurgens* (Walker, 1852)	MI: Mountain St., 46.8692, -87.8933	2019	75	3	M	0.712	0.459
*C. tarsipunctata* (Vorhies, 1909)	MI: Manistee R., 44.2836, -85.8614	2010	100	6	M	0.681	0.409
*C. transversa* (Hagen, 1861)	MN: North Brule R., 48.0076, -90.4169	2001	100	3	M	0.695	0.140
*Leptocerus americanus* (Banks, 1899)	MI: Saint Joseph R., 41.8361, -84.4772	2015	100	6	M	0.235	0.035
*Mystacides interjecta* (Banks, 1914)	MI: Benton Lk., 43.6718, -85.8916	2011	100	3	M	0.321	0.055
*Nectopsyche candida* (Hagen, 1861)	MI: Manistee R., 44.2836, -85.8614	2010	100	3	M	0.594	0.107
*N. pavida* (Hagen, 1861)	IN: Elkhart R., 41.5815, -85.8439	2018	100	3	F	0.254	0.116
*Oecetis avara* (Banks, 1895)	MI: Sturgeon R., 46.5689, -88.6564	2011	100	6	M	0.418	0.135
*O. inconspicua* (Walker, 1852)	MI: Bush Lk., 45.1919, -84.3177	2015	100	6	M	0.453	0.145
*Setodes incertus* (Walker, 1852)	MI: Big Sable R., 44.1176, -86.2010	2014	150	3	M	0.192	0.035
*Triaenodes tardus* Milne, 1934	MN: Bush Lk., 45.1919, -84.3177	2015	50	3	M	0.595	0.166
LIMNEPHILIDAE							
*Anabolia bimaculata* (Walker, 1852)	MI: Silver Lk., 45.2042, -84.3117	2015	15	3	M	2.413	0.531
*A. consocia* (Walker, 1852)	MI: Fairbanks Cr., 44.0481, -85.6586	2011	15	3	M	1.849	0.407
*Hydatophylax argus* (Harris, 1869)	MI: Fairbanks Cr., 44.0481, -85.6586	2010	5	6	F	6.521	1.655
*Limnephilus indivisus* Walker, 1852	MI: Fairbanks Cr., 44.0481, -85.6586	2012	10	3	M	2.295	0.487
*Nemotaulis hostilis* (Hagen, 1873)	MI: Fairbanks Cr., 44.0481, -85.6586	2012	7	3	F	5.515	0.827
*Onocosmoecus unicolor* (Banks, 1897)	MI: Salmon Trout R., 46.8485, -87.7989	2019	25	3	M	2.357	0.604
*Platycentropus radiatus* (Say, 1824)	MI: Fairbanks Cr., 44.0481, -85.6586	2013	8	3	M	3.973	0.596
*Pycnopsyche antica* (Walker, 1852)	MI: Fairbanks Cr., 44.0481, -85.6586	2013	25	6	M	2.263	0.354
*P. guttifera* (Walker, 1852)	MI: Fairbanks Cr., 44.0481, -85.6586	2012	25	6	M	2.199	0.396
*P. lepida* (Hagen, 1861)	MI: Mountain St., 46.8692, -87.8933	2019	25	3	M	2.095	0.342
MOLANNIDAE							
*Molanna blenda* Sibley, 1926	MI: Fairbanks Cr., 44.0481, -85.6586	2011	50	3	M	0.686	0.099
*M. uniophila* Vorhies, 1909	MI: Howe Lk. 46.8932, -87.9436	2019	50	3	M	0.715	0.122
ODONTOCERIDAE							
*Psilotreta indecisa* (Walker, 1852)	MI: Mountain St., 46.8692, -87.8933	2019	50	3	M	0.702	0.179
PHILOPOTAMIDAE							
*Chimarra obscurra* (Walker, 1852)	MI: Livermore Cr., 42.4457, -84.0420	2009	200	6	M	0.354	0.026
*C. socia* (Hagen, 1861)	MI: Sturgeon R., 46.5689, -88.6564	2011	200	6	M	0.402	0.082
*Dolophilodes distinctus* (Walker, 1852)	MI: Fairbanks Cr., 44.0481, -85.6586	2013	100	3	M	0.394	0.067
PHRYGANEIDAE							
*Agrypnia improba* (Hagen, 1873)	MI: Goose Pond, 45.7434, -84.8975	2011	7	3	M	3.059	0.551
*Banksiola crotchi* Banks, 1844	MI: Fairbanks Cr., 44.0481, -85.6586	2013	25	6	M	1.371	0.412
*Phryganea cinerea* Walker, 1852	MI: Fairbanks Cr., 44.0481, -85.6586	2011	5	6	M	6.846	1.504
*Ptilostomis ocellifera* (Walker, 1852)	MI: Fairbanks Cr., 44.0481, -85.6586	2011	5	6	M	7.169	1.367
*P. semifasciata* (Say, 1828)	MI: Slapneck Cr., 46.3331, -86.9369	2011	5	6	M	7.217	2.073
POLYCENTROPODIDAE							
*Holocentropus interruptus* Banks, 1914	MI: Rockwell Lk., 44.0445, -85.6476	2011	50	3	M	0.511	0.982
*Nyctiophylax affinis* (Banks, 1897)	MI: Benton Lk., 43.6718, -85.8916	2011	200	3	M	0.105	0.018
*Polycentropus pentus* Ross, 1941	MI: Fairbanks Cr., 44.0481, -85.6586	2014	75	3	M	0.418	0.092
PSYCHOMYIIDAE							
*Psychomyia flavida* Hagen, 1861	WI: Red R., 44.8022, -88.6711	2015	500	6	F	0.038	0.005
RHYACOPHILIDAE							
*Rhyacophila fuscula* (Walker, 1852)	MI: Miners R., 46.4747, -86.5314	2011	75	6	M	1.402	0.355
SERICOSTOMATIDAE							
*Agarodes distinctus* (Ulmer, 1905)	MI: Howe Lk. 46.8932, -87.9436	2019	25	6	M	0.795	0.127
Thremmatidae							
*Neophylax concinnus* MacLachlan, 1871	MI: Miners R., 46.4747, -86.5314	2019	75	3	M	0.329	0.071

## Discussion

The lack of previous research on the AFDM weights of adult caddisflies renders direct comparisons to other results impossible. Even indirect comparisons are difficult. Of the caddisflies previously weighed via dry mass calculation, none are of the same species that we weighed. Four species: *Agrypnia deflata* (Milne) (Jannot et al. 2007), *Apatania fimbriata* (Pictet) (Wagner 2005), *Mystacides azureus* (L) (Peterson 1989), and *Rhyacophila fasciata* Hagen (Wagner 2005), are within a genus that includes a species that we tested. The four species were 1.3–3.3× heavier than their congeners in our study. Some of that difference is attributable to the different method—dry mass will always be heavier than AFDM because it also includes inorganic matter. Some difference may be due to inherent size difference between congeneric species. Subtle differences in experimental procedure or storage medium may also have led to differences in measured weight. Such differences have frequently been noted in studies of immature aquatic insects (Johnston and Cunjak 1999).

Some weight differences between our specimens and those of other studies may also be due to actual variation between specimens. Several studies have reported 2–5× differences in dry mass between conspecific specimens in the same study due to differences in environmental conditions, larval food quality, or emergence timing (Svensson 1975; Wagner 2002; Wagner 2005). We did not address these topics in our study, instead choosing our specimens based on practical considerations only. Further, our procedure included weighing only one sex per species, weighing specimens of a single collection for each species, and weighing specimens in groups and then calculating standard error based on global means of tested groups. All of these aspects intentionally homogenized biomass variability between specimens. Also, the age of our specimens ranged <1–19 years (Table 1), so some unknown level of decomposition and biomass loss could have taken place in some of the specimens. Thus, our AFDM values should still be considered fairly coarse. Even so, the >500× difference in biomass between the largest and smallest species measured emphasized the increased precision in utilizing AFDM values in ecological calculations instead of simple specimen counting.

**Figure 3.**
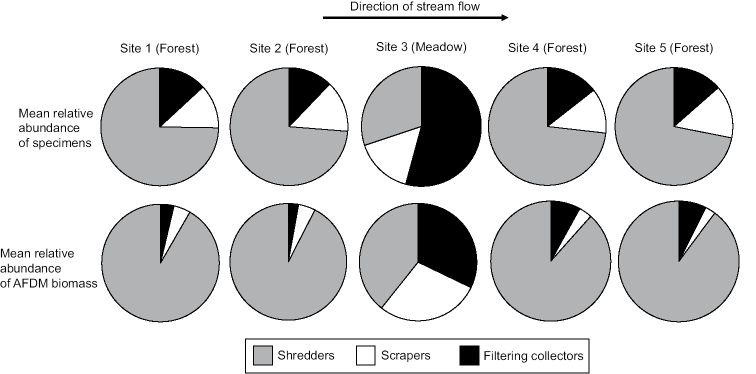
Comparison of mean specimen abundance (% of total specimens) and AFDM biomass (% of total biomass) for the caddisfly FFGs of a Michigan first order stream, based on 13 blacklight samples from each of 5 sites collected weekly from June to August 2012 (Houghton and Wasson 2013). AFDM biomass determined by multiplying determined AFDM values reported herein for each species by the number of specimens of that species in each sample.

This increased precision of using AFDM instead of specimen counting in ecological calculations can be observed when analyzing a previously published data set (Houghton and Wasson 2013). In this study, 13 sets of blacklight samples of adult caddisflies were collected from June to August 2012 at five sites along the continuum of a first order stream in Michigan (USA). The local habitat at the majority of these sites was dense forest, except for a single ~500m stretch of open meadow. The purpose of the study was to assess differences in FFG composition between the forest sites and the meadow site. Based on specimen counting, the authors observed shredders as the dominant FFG at the forested sites, filtering collectors as the dominant FFG at the meadow site, and no change in scrapers throughout the continuum (Figure 3). When substituting the AFDM values per specimen reported herein, biomass of shredders, scrapers, and filtering collectors were approximately equal at the meadow site. This difference is due to the larger body weight of shredders relative to the other FFGs, and the change in dominant scraper taxa along the continuum from the relatively small *Glossosoma nigrior* Banks to the larger *Molanna blenda* Sibley. While not a stark difference from the original conclusions of the study, utilizing AFDM values does allow for a more precise analysis of stream conditions.

These data allow, for the first time, the use of biomass data when assessing stream conditions using adult Trichoptera. Further research will be needed on intra- and inter-population biomass variation within a region. Further, the weights of the fairly high-latitude populations measured in our region may be different than lower latitude populations of the same species. It is our hope that similar studies are conducted in other areas of the US and elsewhere to further increase the value of the adult caddisflies as a biological monitoring taxon.
